# High Efficient and Ultra Wide Band Monopole Antenna for Microwave Imaging and Communication Applications

**DOI:** 10.3390/s20010115

**Published:** 2019-12-23

**Authors:** Shahid Ullah, Cunjun Ruan, Muhammad Shahzad Sadiq, Tanveer Ul Haq, Wenlong He

**Affiliations:** 1 School of Electronic and Information Engineering, Beihang University, Beijing 100191, China; shahidkhan@buaa.edu.cn (S.U.); shahzad_sadiq@hotmail.com (M.S.S.); tanveerulhaq@buaa.edu.cn (T.U.H.); 2 Beijing Key Laboratory for Microwave Sensing and Security Applications, Beihang University, Beijing 100191, China; 3 College of Electronics and Information Engineering, Shenzhen University, Shenzhen 518060, China; wenlong.he@szu.edu.cn

**Keywords:** bandwidth ratio, high efficiency, meander lines, monopole ultra-wideband (UWB) antenna, microwave imaging

## Abstract

The paper presents a highly efficient, low cost, ultra-wideband, microstrip monopole antenna for microwave imaging and wireless communications applications. A new structure (z-shape, ultra-wideband (UWB) monopole) is designed, which consists of stepped meander lines to achieve super-wide bandwidth and high efficiency. Three steps are used to design the proposed structure for the purpose to achieve high efficiency and wide bandwidth. The antenna bandwidth is enhanced by varying the length of meander line slots, optimization of the feeding line and with the miniaturization of the ground width. The simulated and measured frequency bands are 2.7–22.5 GHz and 2.8–22.7 GHz (156% fractional bandwidth), respectively. The dimensions of the antenna are 38 mm × 35 mm × 1.57 mm, and its corresponding electrical size is 2.41 λg × 2.22 λg × 0.09 λg, where guided wavelength λg is at the center frequency (12.75 GHz). This antenna achieved a high bandwidth ratio (8.33:1). The realized gain is varying from 1.6–6.4 dBi, while that of efficiency is 70% to 93% for the whole band. Radiation patterns are measured at four operating frequencies. It has an acceptable group delay, fidelity factor, and phase variation results that satisfy the limit of ultra-wideband in the form of the time domain.

## 1. Introduction

The frequency range of 3.1 GHz to 10.6 GHz band was allocated by federal communication commission (FCC) in 2002 for ultra-wideband application, which is now well-known for both antenna designers and ultra-wideband (UWB) systems [1]. For multiple operating frequencies, multiple antennas installation is difficult due to the limited space within the compact wireless terminal. To solve these problems, researchers designed many novel antennas in the last few years just like reconfigurable antennas [2,3], UWB antennas [4], and multiband antennas. UWB antennas are very useful for the bio-medical purpose such as lung cancer detection and microwave imaging [5,6]. In UWB communication, a compact UWB planar monopole antenna for multiple applications is established. Planar antennas playing a major role among other types of antennas due to its numerous advantages such as its small size, low fabrication cost, simple to design, high flexibility, and easy integration with other devices [7]. During the last few years, many researchers across the globe have been designed planar antennas to achieve wide bandwidth. Among those entire proposed antennas structures, monopole antennas are very popular. In 1976, Dubost and Zisler, first time introduced monopole antennas for wideband application [8].

Monopole antennas have high radiation efficiency, a wider impedance bandwidth, easy in fabrication, wider bandwidth and improved isolation between bands, low profile, low cost and simple in structure [9]. These types of antennas are easily working in wireless personal area networks (WLAN), for high-resolution radars, imaging systems, military communication, cognitive radio, etc. monopole UWB antennas find applications for C-band satellite communication, wireless USB (universal serial bus) dongle, Bluetooth, WiMAX (worldwide interoperability for microwave access) and HIPERLAN/2 [10]. Some of the antennas structures have been proposed with enhanced bandwidth and for multiple applications such as fan-shape [11], spline-shaped monopole [12], U shaped monopole [13], printed T-shape monopole [14], a trident-shaped strip feeding monopole antenna [15], square monopole [16], folded T-shaped element (FTSE) [17] and Koch fractal monopole antenna [18]. A lot of work has been done for wide bandwidth and the miniaturization of the antenna footprint for the commercial and for industrial applications. The properties of impedance matching of monopole antenna feeding lines are very important. Many types of feeding lines have been used to achieve a wide bandwidth such as a microstrip feed slot antenna which having an inverted-F feed line [19], a miniaturized antipodal Y-strip, square slot antenna [20], and monopole antenna with dual orthogonal microstrip feeding lines [21].

Several UWB monopole antennas were designed to achieve wide bandwidth and high efficiency. UWB monopole antenna was designed for wireless personal area networks having 135.2% fractional bandwidth and 79.21% efficiency [22]. In [23] the author designed a valuable antenna having 153.22% fractional bandwidth and 86% efficiency, which will be used for WiMAX/WLAN/ISM (industrial, scientific and medical) and other wireless communication applications. In [24], monopole antenna was designed for wireless communication applications, which have 119.48% bandwidth and 82.22% efficiency. Meander lines structure antennas are also popular for wide bandwidth and high efficiency. In [25], a broadband antenna was designed by using two interconnected meander lines loop, which was operating at 0.55–3.85 GHz frequency bands and got 90.1% high efficiency. In [26], a meander shape monopole antenna was designed to achieve 102% fractional wide bandwidth, which will be used for communication application.

This paper presents a simple z-shaped meander monopole antenna having high efficiency and super ultra-wide bandwidth. It achieved a wide bandwidth of 19.9 GHz (2.8 GHz to 22.7 GHz). A special microstrip feeding line that uses the concept of transformer is used for best matching, and is also good to achieve wide bandwidth. The paper is ordered as follow: Section 2 presents the design of the proposed antenna, Section 3 consists of the parametric study, Section 4 consist operation of the antenna, Section 5 explains the simulation and measured results, Section 6 consist of time-domain analysis, comparison of the proposed work explained in Section 7 and Section 8 is the conclusion of the work.

## 2. Design of Proposed Antenna

**Figure 1:**
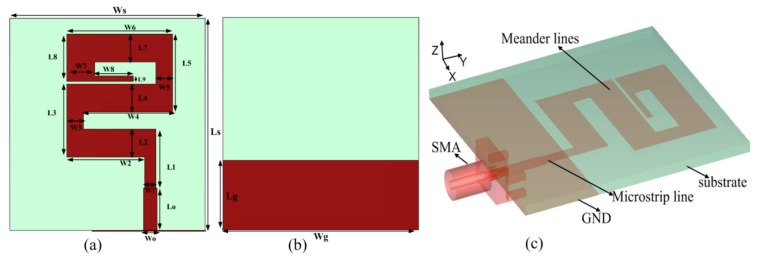
Proposed z-shaped UWB antenna dimensions are: (**a**) Ls = 38 mm, Ws = 35 mm, Wo = 2.4 mm, W1 = 2 mm, W2 = 14 mm, W3 = 3 mm, W4 = 16 mm, W5 = 3 mm, W6 = 19 mm, W7 = 5 mm, W8 = 7 mm, Lo = 7.6 mm, L1 = 10.6 mm, L2 = L4 = L7= 5 mm, L3 = 13 mm, L5 = 14 mm, L8 = 8.5 mm, L9 = 1 mm, (**b**) Lg = 12.5 mm and Wg = 35 mm. Proposed antenna front side (**b**) Left side (**c**) back side which represent DGS, and (**c**) 3D geometry of the antenna.

**Figure 2:**
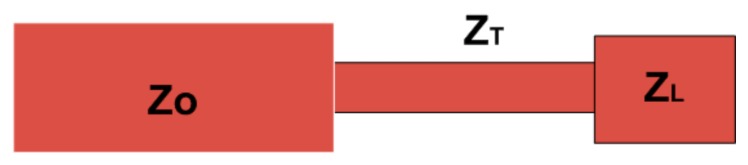
Impedance dimensions of quarter wave transformer and radiating element of the antenna.

The proposed z-shape ultra-wideband monopole antenna has been designed using Roger 5880 substrate having relative permittivity of ε_r_ = 2.2, thickness 1.57 mm and 0.0009 tangent loss as shown in Figure 1. Copper material having a thickness of 0.035 mm is used for ground and as a radiating element of the antenna. According to the theory of monopole antenna, the width and length of the radiating element of the antenna will be λ/100 and λ/4, respectively [13], where “λ” is known as the guided wavelength of the antenna at the center frequency. The dimensions of the antennas are shown in Figure 1. A 70 Ω microstrip line is used as a feeding line of the antenna. This feeding line has used the concept of the quarter wavelength transformer where the feeding line is divided into two parts which have different widths. One of them is a transition line that has a quarter wavelength denoted by width “w_1_” and length “L_1_” as shown in Figure 1a. The proposed quarter-wave transformer is explained in Figure 2. In this figure, “$Z_{T}$” is the transition line impedance which is connecting the radiating meanders line having impedance “$Z_{L}$” with microstrip line having impedance “$Z_{O}$”. The transition line impedance ($Z_{T}$) is calculated with the help of (1) as following [27].

(1) $$Z_{T}=\sqrt{Z_{L}\times Z_{o}}$$

The length and width calculation of transection line is explained in [27,28] and the proposed length and width of the quarter wave transformer feeding line are the optimized values. The calculated impedance of transection line impedance “$Z_{T}$” is 86.4 Ω and meander line impedance “$Z_{L}$” is 77.7 Ω. The dimensions of the microstrip line are shown in Figure 1. The 3-D geometric view of the proposed structure is shown in Figure 1c.

## 3. Parametric Study

This section consists of a parametric study about the proposed structure. Parametric analysis is important to explain about antenna design, about antenna components, dimensions of the components and its effects.

### 3.1. Designing Steps

**Figure 3:**
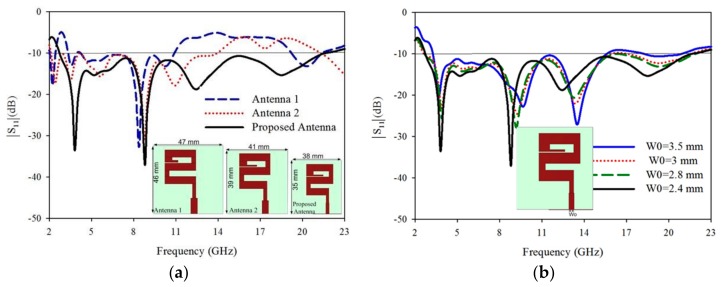
(**a**) Three steps of antenna design and its |S_11_| (dB) results comparison, (**b**) Parametric study of the microstrip line and its |S_11_| (dB) results.

The proposed antenna is designed into three steps, which are shown in Figure 3a along with corresponding |S_11_| (dB) results. At first step antenna, 1 is designed with dimensions 47 mm × 46 mm × 1.57 mm. Some dimensions are the same as shown in Figure 1 and others dimensions are Wo = 4.6 mm, W1 = 2.2 mm, W2 = 18.8 mm, W4 = 18 mm, W6 = 21 mm, W8 = 4 mm, Lo = 10.6 mm, L1 = 9.6 mm, L3 = 14 mm, L9 = 1.5 mm, Lg = 18 mm. The resonance frequencies of this antenna are 2.1–2.41 GHz and 2.9–10.7 GHz. At second step antenna 2 was designed with some of the parameters dimensions are changed such as Wo = 4.2 mm, W2 = 15.8 mm, W4 = 17 mm, W6 = 20 mm, W8 = 6.4 mm, Lo = 8.6 mm, L1 = 7.6 mm, L9 = 1 mm, Lg = 15 mm. The antenna ‘W8′ rod is operating at a lower frequency and the ‘W2” rod is operating at higher frequencies. So when the length of ‘W8′ will be increased then the lower frequency will be decreased and when ‘W2′ length will be decreased than higher frequencies of the band will be increased. This antenna covers 129% fractional bandwidth. At the last step, especially the microstrip line is optimized to achieve wide bandwidth and some other parameter dimensions (W2, W4, W6, and L3) are miniaturized, as shown in Figure 1. The size of these three steps antennas is shown in Figure 3a.

### 3.2. Microstrip Line Width (Wo)

We analyzed the effect of microstrip line width (wo) as shown in Figure 1a. The simulated |S_11_| (dB) results and its variation at different width of the feeding line are as shown in Figure 3b. The width ‘Wo = 2.4 mm’ playing an important role, to get more wide bandwidth. So ‘Wo’ acts as a tuner because when increasing the width then the bandwidth will be narrow and when decreasing the width then bandwidth will be wider.

### 3.3. Effect of Radiating Element (W_2_)

**Figure 4:**
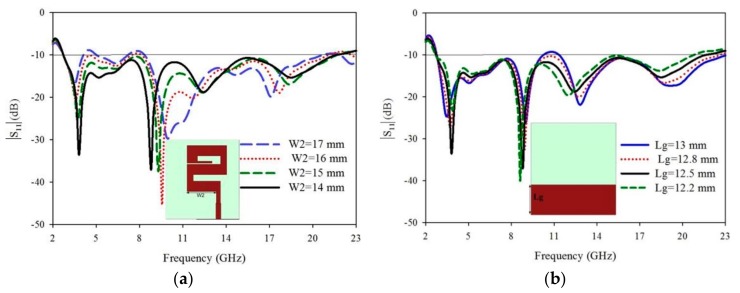
(**a**) Variation of the W2 radiating element and corresponding |S11| results, (**b**) Different dimensions of the ground plane and its corresponding |S11| (dB) results.

Next to the effect of the “W2” radiating element, which dimension is lower than that of the “W4 and W6” as shown in Figure 1. The radiating element ‘W2′ is operating at higher frequencies so according to the antenna designing concept when decreases the length of the antenna then it will operate at a higher frequency and vice versa. The dimensions of ‘W2′ and its |S_11_| (dB) results are shown in Figure 4a.

### 3.4. Effect of Ground Width (Lg)

In monopole antenna, half ground is using which has a greater effect on antenna results. When the part of the ground ‘Lg’ dimensions will more increases or decrease from its limit then it will affect UWB results. In the proposed work the dimension of Lg = 12.5 mm is chosen to get the required result. Different dimensions and it is crossponding |S_11_| (dB) results of the ground are shown in Figure 4b.

## 4. Operation of Antenna

**Figure 5:**
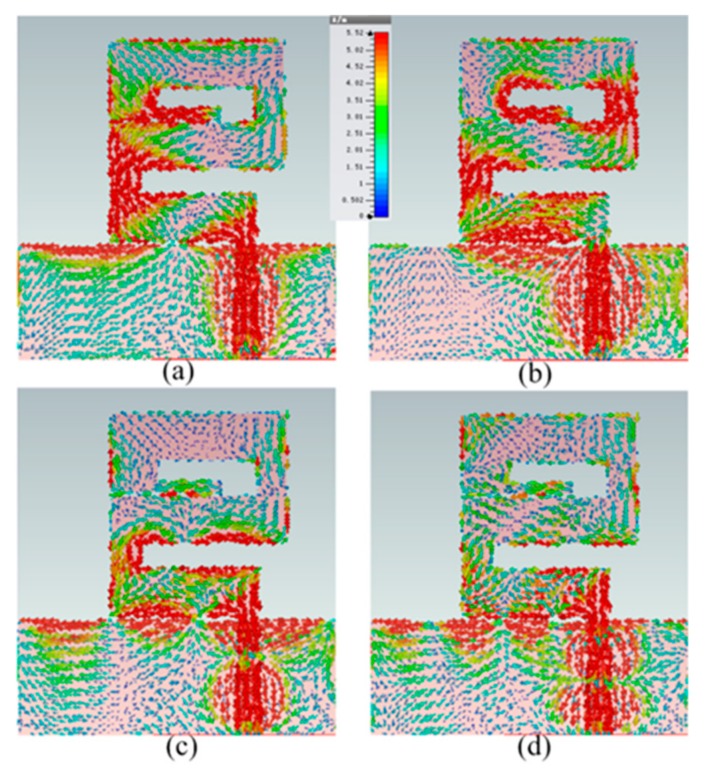
The simulated surface current distribution of the proposed antenna at (**a**) 5.5 GHz (**b**) 8.5 GHz (**c**) 13 GHz (**d**) 20 GHz.

The antenna has been simulated with the help of CST-2015 software. In Section 3.1, it is already discussed that the proposed antenna is simulated and designed in three steps. This section consists of the operation of the antenna. The proposed z-shape antenna consists of interconnected radiating meander lines and every radiating element operating at their own frequencies range. The surface current with the simulation phase angle setup of 0° is shown in Figure 5. The length of the radiating element is dependent on the center frequency of the operating band. The surface current distributions of the proposed antenna corresponding to their operating frequencies are shown in Figure 5. At lower frequencies, the length of the radiating element is greater than that of the higher frequencies. The field of radiation in monopole antennas consists of both the radiator and its ground plane.

The current distribution of the operating frequencies 5.5 GHz, 8.5 GHz, 12.5 GHz, and 20 GHz are shown in Figure 5a–d, respectively. The current distribution field near to the ground field is in the opposite direction because the ground acts as a reflector.

## 5. Simulation and Measured Results

### 5.1. Simulated Results

**Figure 6:**
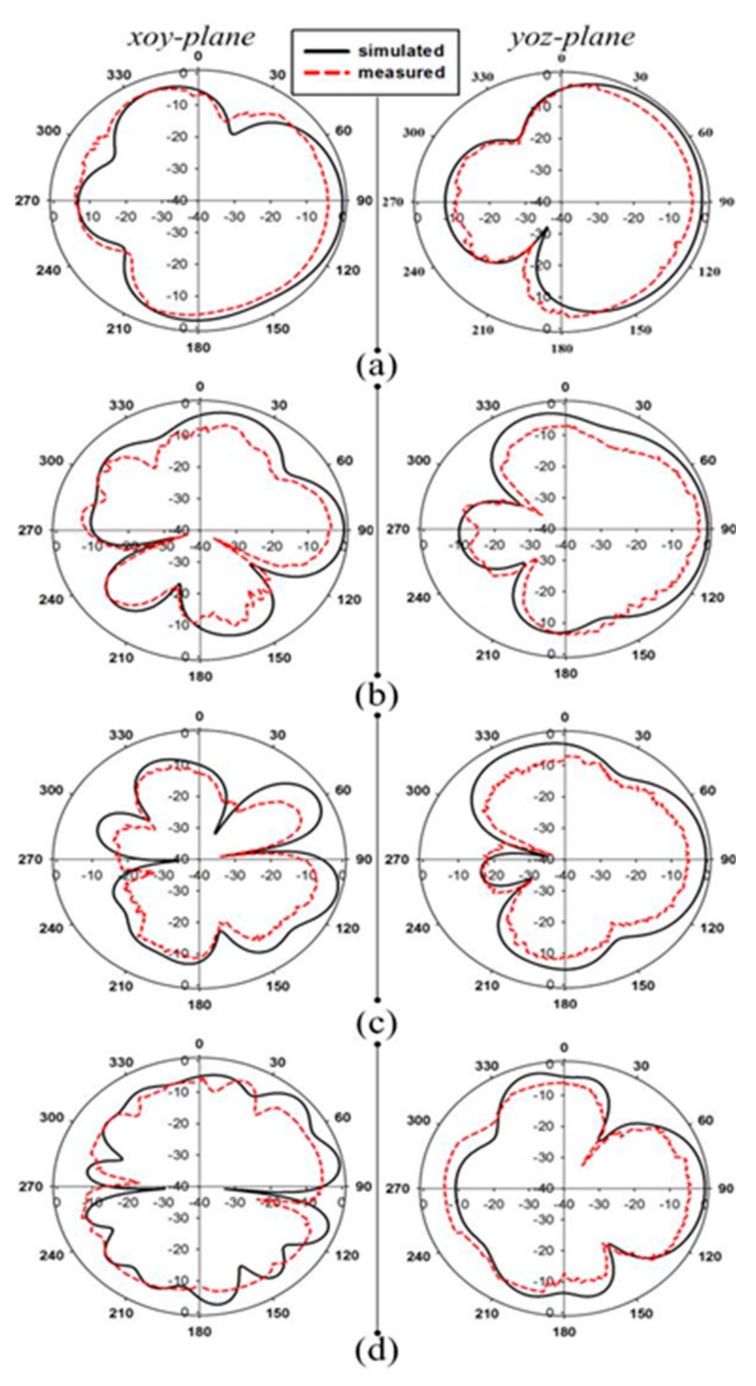
Radiation patterns of the proposed antenna at (**a**) 5.5 GHz (**b**) 8.5 GHz (**c**) 12.8 GHz (**d**) 18 GHz.

**Figure 7:**
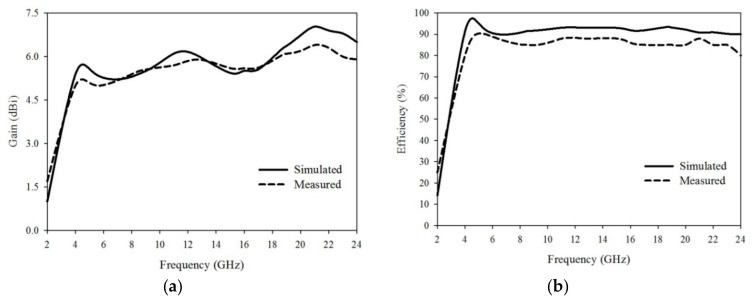
(**a**) Simulated and measured gain of the proposed antenna, (**b**) Simulated and measured efficiency (%) of the proposed antenna.

The proposed antenna simulated (|S_11_| (dB)) result is shown in Figure 3a, and its parametric study is discussed in Section 3. The operating bandwidth of the antenna is from 2.7 GHz to 22.5 GHz, which covers WiMAX(3.45–4 GHz)/ISM(5.725–5.875 GHz)/WLAN(5.15–5.9 GHz)/mobile applications (8.025–8.2 GHz)/defence system (14.62–15.23 GHz) and also passive sensor satellite bands (21.2–21.4 GHz) of the microwave spectrum. The antenna is horizontal polarized and is printed in xy-plane. The E-field of the antenna is in xoy-plane and H-field is in yoz-plane as shown in Figure 6. Due to the large bandwidth only four frequency bands 5.5 GHz, 8.5 GHz, 12.8 GHz, and 18 GHz radiation pattern are shown in Figure 6a–d respectively. The E-field patterns are dumbbell in shape and higher modes are excited at some frequencies, which is clear from the unwanted ripples at the edges of the radiation pattern. H-fields main lobe is in the ‘y-axis’ direction which has end-fire characteristics and nearly Omni-directional. Due to this special characteristic, the antenna is most attractive in microwave imaging and also for communication application. In [6,29] the same characteristics of radiation pattern are explained, which will be used for microwave imaging applications. A maximum of 7.03 dBi simulated gain is achieved at 21 GHz frequency band and the total range of the gain is from 4 dBi to 7.03 dBi as shown in Figure 7a. The antenna is more efficient, as it achieved 97% efficiency at a frequency of 5.5 GHz and also has a constant efficiency above 90% at all the frequencies of the proposed bandwidth as shown in Figure 7b.

### 5.2. Measured Results

The prototype of the antenna is fabricated with the help of a simple thermal transfer method. AutoCAD software, is used to print the shape on thermal paper and heating machine is used to transfer the mask to Rogers 5880. Ferric trichloride (FeCl3) is used for decomposition to etch the layers of black anticorrosion. The proposed antenna has 70 Ω feeding line, so 70 Ω SMA connector will be used but we don’t have this one yet as so we used a special 50 Ω connector part no is (SMA 1-D550B51H01-118) and results are good as mention in the paper.

To measure the radiation patterns, efficiency, and gain of the antenna; the antenna is tested in the anechoic chamber. Measured radiation patterns of xoy-plane (E-field) and yoz-plane (H-field) are shown in Figure 6. The measured results are agreed with the simulated results and small disagreement due to fabrication tolerance and SMA connector. The measured calculated gain and efficiency of the antenna are shown in Figure 7a,b respectively.

**Figure 8:**
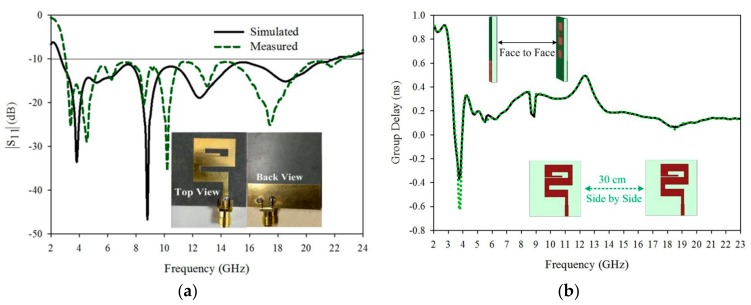
(**a**) Simulated and measured |S11| (dB) results with Proposed antenna prototype, top and back side view, (**b**) Group delay (ns) of the antenna with two different orientations.

To measure |S_11_| (dB) and group delay of the antenna VNA (vector network analyzer), AV3672 is used. The measured S_11_ (dB) and the prototype of the proposed antenna are shown in Figure 8a. The operating measured frequency is from 2.8 GHz to 22.7 GHz and as compared to the simulated result, there are a few shifts that happened in the measured result. The bandwidth of the measure |S_11_| (dB) is also 156% and also covered all the required bands.

## 6. Time Domain Performance

**Figure 9:**
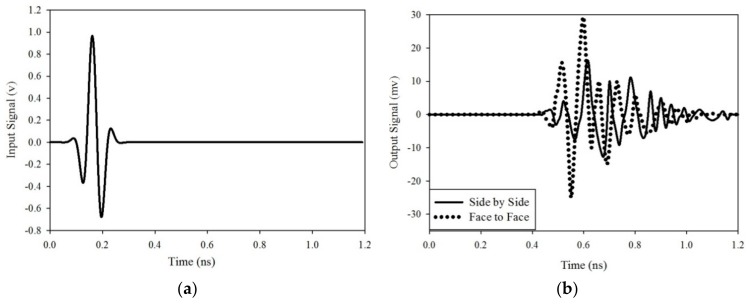
(**a**) Input signals (**b**) output signal for side by side and face to face configuration.

The time-domain behavior of the ultra-wideband antenna is very important to present the performance of the antenna. For this purpose, two identical antennas are placed side by side and face to face at a distance of 30 cm. Group delay (τ) measurement is also important which is the time delay of the transmitting to receiver signal propagation. The proposed antenna group delay (ns) is shown in Figure 8b, for two different configurations. The response of the antenna is good because a less than ±1 ns group delay is detected which is the minimum value and the standard maximum GD (group delay) is Dt = (1/2 fs). The acceptable maximum group delay is 3.8 ns [29]. In the far-field region, one antenna is transmitting signal from one end and another is receiving at the other end. The fidelity factor is the important feature of the wideband antenna, which is calculated with the help of (2) [30].

(2) $$FF=\max_{}\int_{-\infty }^{\infty }S_{i}(t)S_{out}(t+\tau )dt$$

where $S_{i}(t)$ and $S_{out}(t)$ are the input and output signals which are calculated with the help of (3) and (4) respectively. The input and output signals of the proposed antenna are shown in Figure 9, for two different orientations. In it, only the pulses of the signal shape are compared and not its magnitude, as the transmitted signal is much lower than that of the receiving signal.

(3) $$S_{i}(t)=\frac{S_{io}(t)}{{\left[\int_{-\infty }^{\infty }S_{io}^{2}(t)dt\right]}^{\frac{1}{2}}}$$

(4) $$S_{out}(t)=\frac{S_{o}(t)}{{\left[\int_{-\infty }^{\infty }S_{o}^{2}(t)dt\right]}^{\frac{1}{2}}}$$

**Figure 10:**
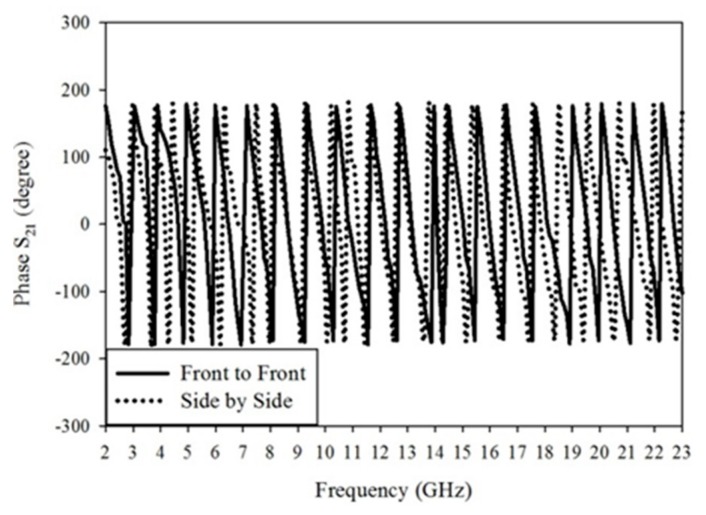
Phase S21 variation with the range of frequencies.

In both of the signals cross-correlation is obtained at every point within time and when both pulses will overlap then the maximum value of correlation will be obtained. The cross-correlation results will be between 0 and 1, due to the signal normalization. When the fidelity factor value will be near to 1 or 1 then it means that input and received signal is identical and less or no dispersion occurring in transmission. If it is zero or near to zero then it means that there is a dispersion in transmission. It means that the fidelity factor value must be greater than 50% (FF > 0.5) [30]. According to the calculation from the given signals, the side by side and face to face fidelity factor values are 0.8065 and 0.8456 respectively. The phase variation plots for side by side and face to face orientation are shown in Figure 10. It shows that the given phase S_21_ there is nearly constant and also linear variations. So with orientation, it has low distortion loss.

## 7. Comparison

The comparison of the proposed antenna bandwidth, gain, size, efficiency, and bandwidth ratio with previous works are shown in Table 1. This table shows that as compared to the previous work the proposed work gets more enhanced results. The concept of the meander line antenna is not new but the proposed Z-shape of the antenna with current specs is new in the research area of monopole UWB antenna which has high efficiency and wide bandwidth. The time-domain performance of the antenna is good which shows that there will be a minimum distortion loss during transmission. The dimensions of the proposed antenna are not miniaturized as compared to the previous works but there is no miniaturized monopole ultra-wideband antenna with such high bandwidth ratio.

## 8. Conclusions

In this work, a simple ultra-wideband and high efficient meander z-shaped monopole antenna has been designed, which covers multiple bands. The size of the antenna is miniaturized with the current bandwidth ratio (8.33:1). The objectives of the antenna are to achieve a high bandwidth ratio and high efficiency. The bandwidth of the antenna is 19.9 GHz which is 156% of the total band. With the help of this antenna, multiple antennas will be replaced into a single antenna. The antenna is more efficient which achieves 93% efficiency. Copper material is used for ground and as a radiator. The radiation patterns are also acceptable but monopole antennas use half ground which has a greater effect on radiation patterns so due to this reason large size metal circuit boards will be not suitable for this antenna. A maximum of 6.4 dBi gains is achieved at 21.5 GHz operating frequency. Due to the high efficiency, ultra-wideband (UWB), reasonable gain, stable radiation pattern, sufficient group delay ensures that the proposed antenna could be applicant for microwave imaging and wireless communication applications.

## Figures and Tables

**Table 1:** Comparison of Proposed Work with Previous Work.

Ref. No’s	Bandwidth (%)	Gain (dBi)	Dimension (mm3)	Efficiency (%)	Lower Frequency (GHz)	Bandwidth Ratio
[2]	105	4.4	25 × 21 × 1.6	90	3.1	3.22:1
[11]	138.3	--	18 × 12 × 1.6	--	2.8	6.2:1
[13]	107.35	4.91	34 × 20 × 1.6	90	2.27	3.3:1
[17]	129.24	3.6	14 × 18 × 1	---	2.94	4.65:1
[18]	122	6	31 × 28 × 1.6	---	3	4.26:1
[22]	135.2	4.85	32 × 32 × 1.6	79.21	2.9	5.17:1
[23]	153	5	25 × 17 × 1.6	86	2.94	7.55:1
[24]	119.48	6.1	35 × 24 × 1.6	82.22	3.1	3.97:1
[31]	138.16	6	32 × 23 × 1.6	---	3.2	5.47:1
[32]	139.88	4.7	25 × 20 × 1.6	---	2.86	5.65:1
[33]	126	6.2	35 × 30 × 0.8	94	2.78	4.42
[34]	138	5.8	50 × 50 × 1.52	88	2.1	5.47:1
Proposed antenna	156	6.4	38 × 35 × 1.57	93	2.7	8.33:1
